# Influence of sampling schedules on [^177^Lu]Lu-PSMA dosimetry

**DOI:** 10.1186/s40658-020-00311-0

**Published:** 2020-06-17

**Authors:** Andreas Rinscheid, Peter Kletting, Matthias Eiber, Ambros J. Beer, Gerhard Glatting

**Affiliations:** 1 Medical Radiation Physics, Department of Nuclear Medicine, Ulm University, Albert-Einstein-Allee 23, 89081 Ulm, Germany; 2 Department of Nuclear Medicine, Ulm University, 89081 Ulm, Germany; 3 Department of Nuclear Medicine, Klinikum Rechts der Isar, Technische Universität München, 81675 München, Germany

**Keywords:** Optimal sampling schedules, Individualized dosimetry, mCRPC, ^177^Lu-PSMA I&T, Single time point, Radioligand therapy

## Abstract

**Background:**

Individualized dosimetry is recommended for [^177^Lu]Lu-PSMA radioligand therapy (RLT) which is resource-intensive and protocols are often not optimized. Therefore, a simulation study was performed focusing on the determination of efficient optimal sampling schedules (OSS) for renal and tumour dosimetry by investigating different numbers of time points (TPs).

**Methods:**

Sampling schedules with 1–4 TPs were investigated. Time-activity curves of the kidneys and two tumour lesions were generated based on a physiologically based pharmacokinetic (PBPK) model and biokinetic data of 13 patients who have undergone [^177^Lu]Lu-PSMA I&T therapy. Systematic and stochastic noise of different ratios was considered when modelling time-activity data sets. Time-integrated activity coefficients (TIACs) were estimated by simulating the hybrid planar/SPECT method for schedules comprising at least two TPs. TIACs based on one single SPECT/CT measurement were estimated using an approximation for reducing the number of fitted parameters. For each sampling schedule, the root-mean-squared error (RMSE) of the deviations of the simulated TIACs from the ground truths for 1000 replications was used as a measure for accuracy and precision.

**Results:**

All determined OSS included a late measurement at 192 h p.i., which was necessary for accurate and precise tumour TIACs. OSS with three TPs were identified to be 3–4, 96–100 and 192 h with an additional SPECT/CT measurement at the penultimate TP. Kidney and tumour RMSE of 6.4 to 7.7% and 6.3 to 7.8% were obtained, respectively. Shortening the total time for dosimetry to e.g. 96 h resulted in kidney and tumour RMSE of 6.8 to 8.3% and 9.1 to 11%, respectively. OSS with four TPs showed similar results as with three TPs. Planar images at 4 and 68 h and a SPECT/CT shortly after the 68 h measurement led to kidney and tumour RMSE of 8.4 to 12% and 12 to 16%, respectively. One single SPECT/CT measurement at 52 h yielded good approximations for the kidney TIACs (RMSE of 7.0%), but led to biased tumour TIACs.

**Conclusion:**

OSS allow improvements in accuracy and precision of renal and tumour dosimetry for [^177^Lu]Lu-PSMA therapy with potentially less effort. A late TP is important regarding accurate tumour TIACs.

## Introduction

In recent years, radioligands labelled with ^177^Lu targeting the prostate-specific membrane antigen (PSMA), such as [^177^Lu]Lu-PSMA-617 [1] and [^177^Lu]Lu-PSMA I&T [2], were established as promising treatment options for patients with metastasized castration-resistant prostate cancer (mCRPC) after exhaustion of approved treatments [3, 4].

Renal dosimetry should be applied for therapy monitoring as kidneys have been identified as a potential dose-limiting organ [2, 5]. First data indicate that additional dosimetry of tumour lesions might predict therapy effect [6]. Inter-patient variabilities in anatomy and (patho‑)physiology can lead to large differences in absorbed dose coefficients. For example, Okamoto et al. reported renal absorbed dose coefficients in the range of 0.33–1.22 Gy/GBq in a cohort of 15 patients with mCRPC treated with [^177^Lu]Lu-PSMA I&T [1]. Thus, individualized treatments are expected to lead to better outcomes than population-based treatments.

The MIRD Pamphlet No. 16 gives suggestions on sampling schedules for individualized dosimetry [7]. At least three time points (TPs) per exponential clearance should be used [7]. Furthermore, one/two TPs at some fraction of the effective half-life, one near the effective half-life and one or two at three and five times the effective half-life were suggested [7]. Thus, individualized dosimetry meeting high accuracy and precision is in general very resource-intensive. Additionally, clinical demand for [^177^Lu]Lu-PSMA radioligand therapy (RLT) is growing. Several studies have already been performed to reduce the number of measurements while maintaining the reliability of the results of dosimetry [8–18]. For example, Merrill et al. investigated sampling schedules comprising 1–3 TPs regarding the time-integrated activity coefficients (TIACs) [19] for patients with Graves’ disease treated with ^131^I [8]. They showed that increasing the number of measurements from two to three only led to marginal improvements in accuracy of the determined TIACs [8]. Dosimetry with a single TP and an assumed effective half-life yielded promising results [8]. In general, dosimetry based on a single measurement was of particular interest (especially for [^177^Lu]Lu-DOTATATE/DOTATOC [13–18]). Therefore, a priori knowledge of the biokinetics, i.e. effective half-lives of the clearance rates, is needed. An elegant single-time-point approach was introduced by Hänscheid et al. [13]. They approximated the TIACs based on the activity value of a single TP. The underlying approximation is exact if the ground truth is mono-exponential and the chosen TP matches the effective half-life. Hänscheid et al. reported that a single measurement at 96 h p.i. led to reliable results of organ and tumour dosimetry for patients with neuroendocrine tumours (NETs) treated with [^177^Lu]Lu-DOTATATE/DOTATOC [13]. Sundlöv et al. and Del Prete et al. used (among others) this single-time-point approach for renal dosimetry and achieved similar accuracies and precisions [14, 15].

The aim of our study was to investigate best accuracy and precision of renal and tumour dosimetry for [^177^Lu]Lu-PSMA I&T therapy with 1–4 TPs. Biokinetic patient data and a physiologically based pharmacokinetic (PBPK) model are used to create time-activity data sets used as ground truths. We seek to identify easy and straightforward sampling schedules optimizing both renal and tumour dosimetry for the hybrid planar/SPECT method using 2–4 TPs and for the single-time-point dosimetry. Additionally, the effects of shortening the time duration for dosimetry on accuracy and precision are systematically investigated.

## Methods

### Patient data and virtual patients

Biokinetic data of 13 patients with mCRPC were obtained by planar whole-body scans at 30–120 min, 24 h and 7 days post injection (additional measurements at 48 h and 72 h p.i. for several patients) for the first cycle [2, 6, 20, 21]. The patient cohort had a median age of 73 years (range: 58–77 years), a median prostate-specific antigen (PSA) level of 133 ng/l (range: 0.23–2905 ng/l) and median kidney and tumour lesion volumes of 297 ml (range: 233–400 ml) and 12 ml (range: 0.33–92 ml), respectively. Activities of 7.3 ± 0.3 GBq [^177^Lu]Lu-PSMA I&T using a peptide amount of 91 ± 5 nmol were applied. Additionally, a pre-therapeutic PET/CT scan with [^68^Ga]Ga-PSMA-HBED-CC (115 ± 16 MBq, 1.6 ± 0.3 nmol) was performed [21].

PBPK modelling was used to create virtual patients. The whole-body PBPK model is described in detail elsewhere [6, 21–23]. In brief, the kidneys, the tumour, the liver and the gastrointestinal tract were modelled as PSMA-positive tissues. The tumour was analysed selecting two tumour lesions (high uptake, no overlap with other PSMA-positive tissue) and a tumour rest. Relevant physically and physiologically mechanisms were included in the PBPK model as e.g. physical decay, blood flows to organs/tumour lesions, specific and unspecific binding, internalisation and release, excretion and plasma protein binding.

The virtual patients were created by individually fitting the PBPK model parameters to the pre-therapeutic PET/CT and the planar biokinetic patient data. Furthermore, individual demographic data were included. Time activity curves (TACs) of the kidneys and the two tumour lesions were generated.

The Ethics Committee of the Technical University Munich approved the retrospective analysis (permit 115/18 S), and the requirement to obtain informed consent was waived.

### Sampling schedules

The simulation routine introduced by Rinscheid et al. [20, 24] was used, which was implemented in MATLAB (release R2019b, The MathWorks, Inc., Natick, MA, USA). The investigated sampling schedules depended on the simulated dosimetric approach. For the hybrid planar/SPECT method, sampling schedules comprising 2–4 planar images and one SPECT/CT measurement were investigated [14, 20, 25–28]. Considering working hours [24], following 24 TPs for planar images were used: 1, 2, 3, 4, 20, 22, 24, 26, 28, 44, 48, 52, 68, 72, 76, 92, 96, 100, 116, 120, 124, 144, 168 and 192 h p.i. For sampling schedules comprising four TPs, the following additional constraint was applied: One or two TPs were within the first 4 h p.i. There were no additional constraints for sampling schedules with two and three TPs. Thus, 276, 2024 and 5700 different sampling schedules for the planar images were investigated comprising of 2, 3 and 4 TPs, respectively. For the hybrid planar/SPECT method, the quantitative SPECT/CT measurement was assumed to be 0.5 h after one of the planar images of the investigated sampling schedules. This resulted in 2 × 276, 3 × 2024 and 4 × 5700 sampling schedules for the hybrid planar/SPECT method comprising of 2, 3 and 4 planar images, respectively. For the single-time-point approach [13], each TP considered for planar images were investigated for the time of the single SPECT/CT scan (i.e. 24 cases).

### Time-activity data sets

Ground truths, i.e. time-activity curves *A*_true_(t) of the kidneys and of two tumour lesions, were generated from the virtual patients. Thus, the true activity values for each sampling schedule are known. Random noise was taken into account for each activity value. The used noise model is described in detail in the supplement. In brief, the simulated activity values *A*_planar_(*t*_*i*_) and *A*_SPECT_(*t*_SPECT_) were randomly drawn from log-normal distributions [29]. The standard deviations of the distributions depended on the imaging modality (planar: 20 %; SPECT/CT: 5 %) [20]. The noise of activity values attributed to planar images was subdivided into a systematic and a stochastic part [30], i.e. a superposition of two log-normal distributions was used. The amount of systematic noise (*f*_syst_) in the total noise (20 %) is an unknown parameter, which depends e.g. on the anatomy of the patient, the measurement device and the quantification process [30]. Thus, different proportions *f*_syst_ = 25%, 50% and 75% were investigated for the hybrid planar/SPECT method [20].

### Time-integrated activity coefficients with the hybrid planar/SPECT approach

For determining the TIACs with the planar/SPECT approach, the simulated planar activity values *A*_planar_(*t*_*i*_) were firstly fitted with a mono-exponential function:

1 $$ {f}_{\mathrm{planar}}(t)={A}_1\cdot {\mathrm{e}}^{-\left({\lambda}_1+{\lambda}_{\mathrm{phys}}\right)\cdot t} $$

with the prefactor *A*_1_, the biological clearance rate *λ*_1_ and the physical decay constant for ^177^Lu *λ*_phys_ = ln(2)/(6.647 ∙ 24) h^−1^ [31]. The TIACs based on the planar images (*TIAC*_planar_) were determined by analytical integration of *f*_planar_ from zero to infinity and subsequent normalization as

2 $$ {TIAC}_{\mathrm{planar}}=\frac{1}{A_0}\cdot {\int}_0^{\infty }{f}_{\mathrm{planar}}(t)\mathrm{d}t=\frac{1}{A_0}\cdot \frac{A_1}{\lambda_1+{\lambda}_{\mathrm{phys}}} $$

where *A*_0_ is the injected activity for the investigated patient. The TIACs estimated with the hybrid planar/SPECT method (*TIAC*_hybrid_) were calculated according to

3 $$ {TIAC}_{\mathrm{hybrid}}=\frac{A_{\mathrm{SPECT}}\left({t}_{\mathrm{SPECT}}\right)}{f_{\mathrm{planar}}\left({t}_{\mathrm{SPECT}}\right)}\cdot {TIAC}_{\mathrm{planar}} $$

where *A*_SPECT_(*t*_SPECT_) is the simulated activity value assuming the SPECT/CT measurement and *f*_planar_(*t*_SPECT_) is the activity value according to the fit function used for fitting the planar activity data set at TP *t*_SPECT_ [14, 20, 28]. The relative differences Δ of the simulated TIACs and the ground truth were determined. The values of Δ*TIAC* also correspond to the relative differences in self-doses.

### Time-integrated activity coefficients with single time point approach

The dosimetry method introduced by Hänscheid et al. [13] with just one single quantitative SPECT/CT measurement was investigated. The TIACs can be approximated as

4 $$ {TIAC}_{1\mathrm{TP}}=\frac{1}{A_0}\cdot \frac{2}{\mathrm{In}(2)}{A}_{\mathrm{SPECT}}\left({t}_{\mathrm{ref}}\right)\cdot {t}_{\mathrm{ref}} $$

where *A*_SPECT_(*t*_ref_) is the simulated activity value for the SPECT/CT measurement at TP *t*_ref_ [13, 14].

### Optimal sampling schedules

In total, 1000 replications were performed for each sampling schedule and patient [24]. Thus, 13000 Δ*TIAC* values for the kidneys and 26000 Δ*TIAC* values for the tumours were simulated for each sampling schedule. The mean (*μ*_Δ*TIAC*_) and standard deviation (*σ*_Δ*TIAC*_) of the Δ*TIAC* values were used to estimate the root-mean-squared error RMSE for the kidneys (*RMSE*_K_) and tumours (*RMSE*_T_) individually according to

5 $$ {RMSE}_j=\sqrt{{\left({\sigma}_{\Delta TIAC,j}\right)}^2+{\left({\mu}_{\Delta TIAC,j}\right)}^2} $$

where the index *j* represents the number of the sampling schedule. Lower RMSE values represent better sampling schemes for the kidneys or tumour lesions. A joint *RMSE*_joint_ value was introduced to sort the sampling schedules with respect to accurate and precise results for the kidneys and tumours:

6 $$ {RMSE}_{\mathrm{joint},j}={w}_K\cdot {RMSE}_{\mathrm{K},j}+{RMSE}_{\mathrm{T},j\cdot } $$

where *w*_K_ is a weighting factor for the kidney RMSE. A weighting of *w*_K_ = 2 was used for the simulations to ensure a higher priority of accurate and precise kidney dosimetry than tumour dosimetry. The schedule with the lowest *RMSE*_joint_ values was defined as the optimal sampling schedule (OSS).

The effect of varying the last two TPs of the determined OSS on the kidney *RMSE*_K_ and on the tumour *RMSE*_T_ was investigated for the hybrid planar/SPECT method. Additionally, the best achievable RMSE by limiting the time of the last measurement to 48 h, 72 h, 96 h,… and 192 h were estimated.

## Results

### Optimal sampling schedules

The determined OSS for estimating renal and tumour TIACs using the hybrid planar/SPECT method were independent on the investigated fraction of systematic error *f*_syst_ to the total error with one exception (Table 1). The RMSE values decrease with increasing *f*_syst_ for a fixed total error. This shows that the systematic error due to planar images can be mostly corrected using the hybrid planar/SPECT method. Higher RMSE values were found for tumours than for the kidneys. This is particularly evident for the sampling schedules comprising two TPs. Best achievable RMSE values of either only the kidneys or the tumours resulted in lower RMSE values of maximal 0.6 percentage points (data not shown) than joint optimization. Thus, a joint optimization of the kidneys and tumours was possible using 2–4 TPs and the hybrid planar/SPECT method. The method with a single SPECT/CT resulted in kidney *RMSE*_K_ values similar to those estimated with 2–4 TPs and the hybrid planar/SPECT method. The tumour TIACs were considerably underestimated.

**Table 1:** OSS for joint kidney and tumour dosimetry based on the hybrid planar/SPECT method and on the approximation by Hänscheid et al. [13] (Eq. 4)

*f*_syst_^a^ (%)	*N*_TP_^b^	*t*_SPECT_^c^	OSS^d^ (h)	*RMSE*_K_^e^ (%)	*μ*_K_ ± *σ*_K_^f^ (%)	*RMSE*_T_^e^ (%)	*μ*_T_ ± *σ*_T_^f^ (%)
―^g^	1	*t*_1_	**52**	7.0	− 2.8 ± 6.4	16	− 14 ± 7.6
25	2	*t*_1_ + 0.5 h	**20**, 192	8.0	0.6 ± 8.0	13	− 0.2 ± 13
50	2	*t*_1_ + 0.5 h	**20**, 192	7.4	0.5 ± 7.4	11	− 0.6 ± 11
75	2	*t*_1_ + 0.5 h	**20**, 192	6.6	0.2 ± 6.5	9.0	− 1.0 ± 8.9
25	3	*t*_2_ + 0.5 h	3, **96**, 192	7.7	− 1.0 ± 7.7	7.8	− 1.9 ± 7.5
50	3	*t*_2_ + 0.5 h	3, **96**, 192	7.1	− 1.0 ± 7.0	7.1	− 2.2 ± 6.8
75	3	*t*_2_ + 0.5 h	4, **100**, 192	6.4	− 0.7 ± 6.3	6.3	− 1.8 ± 6.0
25	4	*t*_3_ + 0.5 h	3, 4, **92**, 192	7.2	− 1.1 ± 7.1	7.6	− 1.4 ± 7.5
50	4	*t*_3_ + 0.5 h	3, 4, **92**, 192	6.6	− 1.3 ± 6.5	6.9	− 1.7 ± 6.7
75	4	*t*_3_ + 0.5 h	3, 4, **92**, 192	5.9	− 1.3 ± 5.8	6.3	− 2.0 ± 6.0

^a^Fraction of systematic error to the total error in the planar images

^b^Number of time points

^c^Time point of the SPECT/CT

^d^Optimal sampling schedules

^e^Root mean-squared error of the kidneys (subscript “K”) and tumours subscript (“T”)

^f^Mean and standard deviation of the relative deviations of the simulated time-integrated activity coefficients from the ground truth

^g^No division into systematic/stochastic error needed (simulated noise for the SPECT/CT: 5 %)

Dosimetry with 2–4 planar images only, i.e. without a SPECT/CT measurement, led to kidney and tumour RMSE values about two to three times higher than with the hybrid planar/SPECT method (exceptions for the tumour *RMSE*_T_ values using two TPs and *f*_syst_ = 25 %, 50 %; 1.4- and 1.7-fold). The determined OSS for dosimetry based on planar images only are listed in Additional file 1: Table S1.

**Fig. 1:**
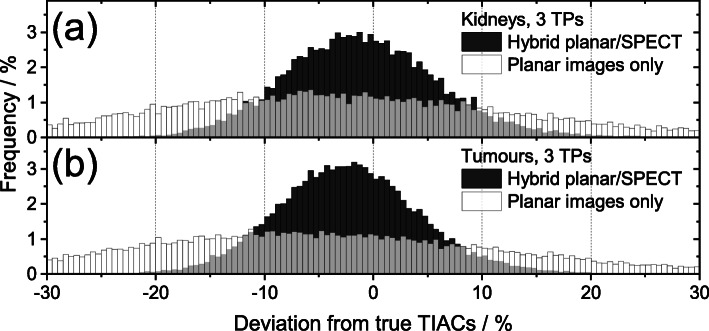
Comparison of the deviations between the simulated TIACs and the ground truths considering **a** the kidneys and **b** the tumours using the hybrid planar/SPECT method (dark grey) and planar images only (white) with three time points (TPs). For the hybrid planar/SPECT method, the TIACs were determined using the optimal sampling schedule of 3, 96 and 192 with *t*_SPECT_ = *t*_2_ + 0.5 h (Table 1). The used optimal schedule for dosimetry based on planar images was 20, 28 and 192 h (Additional file 1: Table S1). The systematic error of the simulated activity values based on planar images was assumed to contribute 50% to the total error

The frequency distributions of the relative deviations between the simulated TIACs and the ground truths for the hybrid planar/SPECT method and for the planar images only are presented in Fig. 1. The broad frequency distribution of the simulated kidney TIACs based on the planar images e.g. led to about 25% of the TIACs deviating more than 20% from the ground truth. This percentage was reduced below 1% by using the hybrid planar/SPECT method instead.

**Fig. 2:**
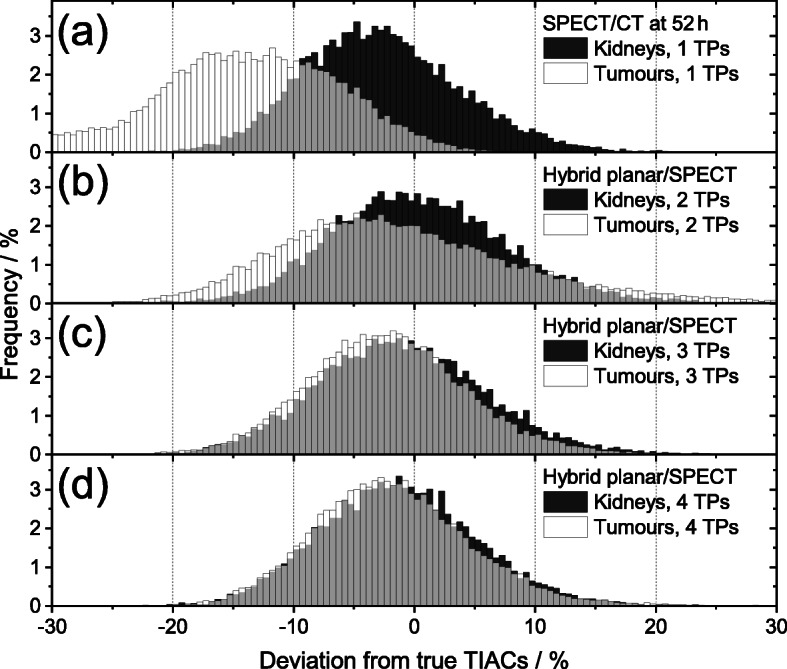
Comparison of the frequency distribution of the deviations between the simulated TIACs and the ground truths using **a** the method with one SPECT/CT at 52 h and the hybrid planar/SPECT method with optimal sampling schedules comprising **b** two time points (TPs), **c** three TPs and **d** four TPs (Table 1). The kidneys (dark grey) and the tumours (white) of all 13 patients were considered. The systematic error of the simulated activity values based on planar images was assumed to contribute 50% to the total error

The frequency distributions of the kidneys and tumours using 1–4 TPs are depicted in Fig. 2. One single SPECT/CT measurement at 52 h resulted in a high number of underestimations of the tumour TIACs (Fig. 2a). A more appropriate TP regarding only tumour dosimetry was determined to be 72 h leading to the kidney and tumour RMSE values of 11 % (mean: − 6.1 ± 9.1%) and 12% (mean: − 7.8 ± 8.8%), respectively. Using two TPs led to a skewed distribution of the tumour TIACs (Fig. 2b). Thus, the standard deviations of the kidneys *σ*_K_ and the tumours *σ*_T_ differed (Table 1) by about 30–60%. Using three and four time points resulted in similar frequency distributions for kidney and tumour TIACs, which can also be seen from the similar means *μ* and standard deviations *σ* given in Table 1.

### Variation of the last two time points from the determined optimal sampling schedule

**Fig. 3:**
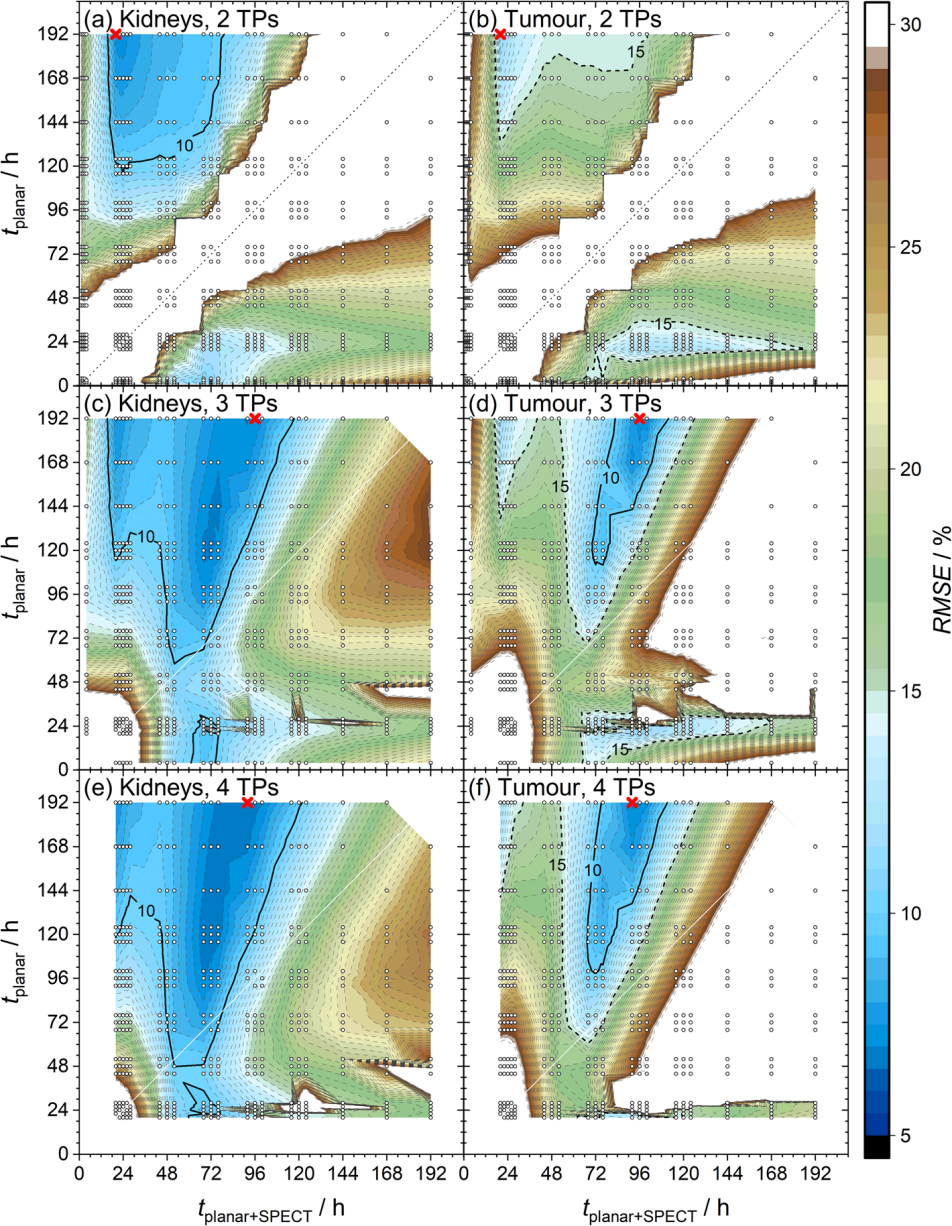
Effect of varying the last two time points (time point of the planar image followed by the SPECT/CT: *t*_planar + SPECT_) from the optimal schedules for the hybrid planar/SPECT method (Table 1) on the root-mean-square error (RMSE) values of the kidneys and the tumours. The optimal schedules and the investigated alternatives of the last two time points are marked with red crosses and white circles, respectively. Contour lines with *RMSE* = 10 % for the kidneys (black line) and with *RMSE* = 10%, 15% (black dashed line) for the tumours are highlighted. The results base on the simulations using a fraction of systematic error to the total error of *f*_syst_ = 50%

The dependence of the RMSE on variations of the last two TPs from the determined OSS for the hybrid planar SPECT/CT method (Table 1) was investigated (Fig. 3).

The dependence of the RMSE on the sampling schedule comprising *two TPs* are depicted in Fig. 3a, b. A reduction of the total time for dosimetry with acceptable accuracy and precision (e.g. kidney *RMSE*_K_ < 10% and tumour *RMSE*_T_ < 15%) could be achieved with sampling schedules in the range of 20–24, 144h (*t*_SPECT_ = *t*_1_ + 0.5 h). Furthermore, the schedule of 4 and 68 h (*t*_SPECT_ = *t*_2_ + 0.5 h) seemed promising. However, the kidney RMSE was slightly above 10% (*RMSE*_K_ = 11%, *RMSE*_T_ = 14%). Here, the *RMSE*_T_ value increased with decreasing *t*_1_ from 4 to 1 h.

Investigations of variations from the determined sampling schedule comprising *three TPs*, i.e. 3, 4–168 and 20–192 h (*t*_SPECT_ = *t*_2,3_ + 0.5 h), are depicted in Fig. 3c, d. RMSE values below 10 % for both kidneys and tumours could be reached within 120 h using e.g. 3, 72 and 120 h (*t*_SPECT_ = *t*_2_ + 0.5 h). Here, the kidney *RMSE*_K_ = 6.9 % was even slightly improved in comparison to the joint OSS. Dosimetry within 72 h by e.g. using 3, 20 and 72 h (*t*_SPECT_ = *t*_3_ + 0.5 h) as sampling schedule led to kidney *RMSE*_K_ = 9.3% and tumour *RMSE*_T_ = 13%.

Figure 3e, f shows the RMSE in dependence of the used sampling schedules comprising *four TPs* within 3, 4, 20–168 and 22–192 h (*t*_SPECT_ = *t*_3,4_ + 0.5 h). The time duration for dosimetry could also be shortened to 120 h with both RMSE values still below 10%. Dosimetry within 72 h was possible with kidney *RMSE*_K_ = 8.2% and tumour *RMSE*_T_ = 13% using e.g. 3, 4, 68 and 72 h (*t*_SPECT_ = *t*_3_ + 0.5 h). These RMSE values could be further reduced to *RMSE*_K_ = 8.0% and *RMSE*_T_ = 11% by e.g. using 4, 20, 68 and 72 h (*t*_SPECT_ = *t*_3_ + 0.5 h; data not shown).

The effects on the RMSE by varying the last two TPs of the determined OSS for the simulations with *f*_syst_ = 25 % and *f*_syst_ = 75 % are given in the supplement (Additional file 1: Figures S1 and S2).

### Reductions of the time duration for dosimetry

**Fig. 4:**
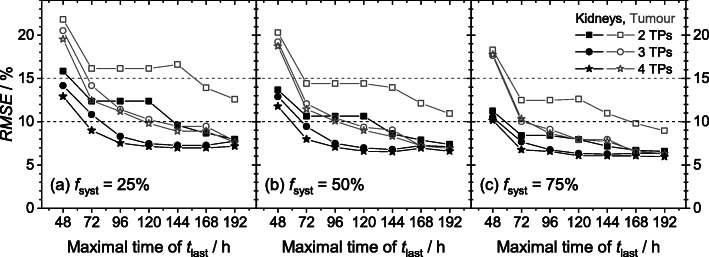
Best achievable root-mean-squared error (RMSE) values as a function of the latest used measurement time *t*_last_ for different fractions of systematic error *f*_syst_ of **a** 25%, **b** 50% and **c** 75%. The RMSE of the kidneys (filled black) and the tumours (open grey) for different number of time points TPs (2: square; 3: circle; 4: star) are depicted. The horizontal lines represent *RMSE* = 10 % (black dashed) and *RMSE* = 15% (grey dashed) representing the ad hoc assumed limits for the kidneys and tumours, respectively

The best achievable RMSE by using the hybrid planar/SPECT method with limiting the time for the last TP *t*_last_ of the sampling schedules are depicted in Fig. 4. Only slight changes (≤ 1.0 percentage points) of the kidney RMSE was observed for OSS comprising three or four TPs with *t*_last_ = 96 h…192 h. For tumours and investigated schedules with three and four TPs, the RMSE steadily increased with shortening time duration for dosimetry, i.e. with decreasing *t*_last_. Using four instead of three TPs resulted in lower RMSE values of less than 0.8 percentage points for *t*_last_ = 96 h…192 h. The schedules 4, 68–72 and 96 h (*t*_SPECT_ = *t*_2_ + 0.5 h) were best suited for dosimetry within 96 h p.i. Dosimetry within 72 h with kidney *RMSE*_K_ ≤ 10% and tumour *RMSE*_T_ ≤ 15% was possible using schedules with four TPs for *f*_syst_ = 25%, with at least three TPs for *f*_syst_ = 50% and even with two TPs for *f*_syst_ = 75%. Dosimetry with *RMSE*_K_ ≤ 10% and *RMSE*_T_ ≤ 15% within 48 h was not possible.

## Discussion

Individualized dosimetry for PSMA targeting agents labelled with ^177^Lu is demanding high resources especially when high accuracy and precision are required. Simplified dosimetric approaches leading to reliable results are therefore needed. In this study, the achievable accuracy and precision (combined in the RMSE) for the kidney and tumour TIACs in [^177^Lu]Lu-PSMA I&T therapy were investigated. The hybrid planar/SPECT method and the method introduced by Hänscheid et al. using one single SPECT/CT scan [13] were used. OSS for joint renal and tumour dosimetry comprising four TPs (3, 4, 92, 192 h), three TPs (3–4, 96–100, 192 h), two TPs (20, 192 h) and one single TP (52 h) were identified. For the hybrid planar/SPECT method (2–4 TPs), the SPECT/CT was assumed to be 0.5 h after the penultimate planar measurement in all cases. As all these OSS have a very late TP, the effects of shortening the time duration for dosimetry on the RMSE was additionally investigated. Dosimetry with one single SPECT/CT at 52 h p.i. yielded promising results for kidney TIACs, but biased tumour TIACs.

The renal and tumour RMSE values were similar considering three and four optimized TPs with at least one TPs ≥ 96 h (Figs. 3c–f and 4). Thus, three TPs may be sufficient for accurate and precise renal and tumour dosimetry using a mono-exponential fit function. To account for practicability in clinical routine and patient comfort, the sampling schedule of 4, 68–72 and 96 h (*t*_SPECT_ = *t*_2_ + 0.5 h) can be proposed as a suitably shortened OSS.

OSS with three TPs for renal dosimetry alone were already determined earlier [20]. There, we showed that using a schedule of 3–4, 72–76 and 124–144 h p.i. with a SPECT/CT at *t*_2_ + 0.5 h led to renal RMSE of 6.2 –7.2 %. These results were reproduced within this study as shown in Fig. 3c and Additional file 1: Figures S1c and S2c. Thus, TPs later than about 144 h p.i. were not necessary for renal dosimetry alone. All determined optimal sampling schedules for joint renal and tumour dosimetry comprised a late TP at 192 h. This late TP was therefore important for additional accurate and precise tumour TIACs as shown in Fig. 3d.

Using planar images at 4 h and 68 h with a SPECT/CT following the last measurement or a single SPECT/CT measurement at 52 h p.i. yielded good results for the estimation of renal TIACs. These approaches are expected to be sufficient if additional accurate tumour dosimetry is not required. In our study, dosimetry based on the hybrid planar/SPECT method seems to outperform dosimetry based on planar images only, even if fewer time points were used.

The single SPECT/CT measurement for treatment control might be pre-defined in nuclear medicine departments based on their individual logistics. Therefore, the time point of the SPECT/CT scan may be chosen different to the determined optimal time point. Assuming the SPECT/CT scan defined at e.g. 24 h p.i., a final planar image at about 144–168 h p.i. should be considered if accurate and precise tumour dosimetry is of interest (Fig. 3). In any case, our simulations allow estimating the loss of accuracy and precision due to a pre-defined SPECT/CT measurement.

Aiming at dosimetry with a single TP, inclusion of a priori knowledge is essential. The here used approximation of the TIACs with Eq. 4 would be exact if the ground truth is a mono-exponential function, and the time of the single measurement matches the effective half-life [13] (i.e. the used a priori knowledge is the effective half-life). Since the kidneys and the tumour lesions have different effective half-lives, the joint optimisation with this dosimetric approach did not lead to satisfactory tumour dosimetry. A more suitable approach might e.g. be the usage of averaged population values of the effective half-lives depending on the investigated organs and tumours [12]. This procedure was not investigated in this study.

Several groups have already investigated a single-time-point approach on patients with neuroendocrine tumours (NETs) and meningioma injected with [^177^Lu]Lu-DOTATATE/DOTATOC [13–15]. The used TPs and the deviations from the respective ground truths are listed in Table 2. In this study, using a single TP at 96 h p.i. mostly underestimated the renal absorbed dose. This has not been observed that drastically in the literature [13–15]. These differences can have several causes. Firstly, different tumour entities and radiopharmaceuticals have been investigated (mCRPC vs. NETs/meningioma; [^177^Lu]Lu-PSMA I&T vs. [^177^Lu]Lu-DOTATATE/DOTATOC). Secondly, the kidneys showed different effective half-lives. For NETs and meningioma patients treated with [^177^Lu]Lu-DOTATATE/DOTATOC, effective half-lives of 47–52 h are given for the kidneys in the literature [13–15]. In this study, a median effective half-life of the kidneys of 40 h (range: 30–62 h) and of the tumours of 50 h (range: 34–94 h), respectively, from 24 h onwards was determined. Thirdly, the ground truths from the literature based on mono-/bi-exponential functions fitted to the full-time activity data sets. In contrast, a whole-body PBPK model was used to create the ground truth in this study. The results provided by Hänscheid et al. [13] using a single measurement at 48 h p.i. were more consistent with our results considering the determined OSS at 52 h p.i.

**Table 2:** Deviations of renal absorbed doses using one single measurement for [^177^Lu]Lu-PSMA I&T (this study) and [^177^Lu]Lu-DOTATATE/DOTATOC (literature)

	This study		Hänscheid et al. [13]		Sundlöv et al. [14]	Del Prete et al. [15]
Time point	52 h	96 h	48 h	96 h	96 h	96 h
Minimum	− 26 %	− 48 %	− 33 %	− 9 %	n/a^b^	− 38 %
0.1 quantile	− 11 %	− 36 %	− 8 %	− 3 %	n/a^b^	− 0.4 %
Median	− 3.1 %	− 17 %	0 %	5 %	n/a^b^	5.8 %
0.9 quantile	5.7 %	0.5 %	9 %	10 %	n/a^b^	9.2 %
Maximum	24 %	21 %	17 %	17 %	n/a^b^	17 %
Mean	− 2.8 %	− 18 %	n/a^b^	n/a^b^	1 %	n/a^b^
1 × *σ*^a^	6.4 %	14 %	n/a^b^	n/a^b^	5.5 %^c^	n/a^b^

^a^Standard deviation

^b^Not available

^c^Provided result: 2 × *σ* = 11%

A weighting factor of *w*_k_ = 2 was used for the kidney RMSE values (Eq. 6). Using weightings *w*_k_ of e.g. 1 and 4 only had minor effects on single time points of the OSS with 2–4 time points (± 1 h for *t*_*i*_ ≤ 4 h and ± 4 h for *t*_*i*_ ≥ 20 h). For the single-time-point approach, a weighting factor of *w*_k_ = 1 led to an OSS of 68 h, which was more favourable for tumour dosimetry. Factors with *w*_k_ ≥ 2 did not further change the OSS of 52 h for the single time point approach, i.e. it was already optimized for renal dosimetry.

The simulation routine used a mono-exponential fit function for all-time activity data. A fit function neglecting an initial uptake phase seems an acceptable simplification for the kidneys and for the tumour lesions [7]. Regarding kidney kinetics, the median maximum TAC value was at 2 h (range: 0.6–3 h). Furthermore, at least 96.8% of the maximum kidney’s activity value was reached already 1 h post injection in all virtual patients. The tumour lesions showed slower uptake kinetics compared to the kidneys. Here, the median maximal activity uptake value was reached after 2.5 h (range: 0.5–9 h). Three hours post injection an uptake value of at least 95% was reached in almost all investigated tumour lesions (two exceptions with 89% and 86%). Nevertheless, using a set of appropriate fit functions and selection criteria [32, 33] could further improve accuracy and precision. Clearly, different optimal sampling schedules are expected for other fit functions.

Noise levels of 5% for SPECT/CT and 20% for planar measurements seem reasonable for kidney activity values [20]. For simplicity, the same noise levels were used for simulated activity values in tumour lesions. However, higher noise might be in general more realistic for small tumour lesions [24]. Furthermore, the noise levels were assumed to be constant over time. Obviously, this is an approximation as e.g. Poisson noise increases for later time points. Assuming a minimal kidney activity at 192 h p.i. of 3.1 MBq, a sensitivity of 9.4 cps/MBq [34], a field-of-view in *z*-direction of 38.7 cm and a bed speed of 10 cm/min, a maximal Poisson noise of 1.2% can be expected for the measured counts within the kidney ROIs for planar imaging. Thus, increases in Poisson noise over time could be neglected for the kidneys. For the tumour lesions, the total uptake of activity and thus the Poisson noise is size dependent. Here, in analogy a median Poisson noise of 1.1% (range: 0.3–9.2%) can be estimated at 192 h p.i. for planar imaging, where the maximal Poisson noise came from the smallest investigated tumour lesion of 0.33 ml volume. Thus, higher noise levels of about 25–30 % could have been more realistic for small tumour lesions with low activity uptake for late time points. We expect that this would generally lead to higher RMSE values for the tumour lesions. Furthermore, sampling schedules with a last time point earlier than 192 h might be more favourable for tumour dosimetry.

Individual pre-therapeutic PET/CT and planar imaging data were used to estimate the PBPK model parameters. Clearly, quantitative SPECT/CT data instead of planar images would additionally improve the estimations of the parameters. Thus, differences between the virtual patients’ biokinetics used as ground truth and the true patients’ biokinetics (which is unknown) may exist. However, since a population of virtual patients with different uptake and washout kinetics was investigated, we expect only minor changes of the results with SPECT/CT input data. Nevertheless, the determined OSS have to be validated in future prospective studies.

## Conclusion

The used simulation routine is ideally suited to determine optimal sampling schedules for combined renal and tumour dosimetry in [^177^Lu]Lu-PSMA I&T therapy. Considering 2–4 time points, best results are achieved with a last time point at 192 h p.i. The difference in accuracy and precision between optimal sampling schedules with three and four TPs is marginal. Thus, dosimetry based on not more than three time points seems to be sufficient. Focusing on renal dosimetry only, the overall time duration of dosimetry can be safely shortened to e.g. 96 h p.i. when using three time points. Dosimetry based on one single time point at 52 h p.i. led to reliable renal TIACs but biased tumour TIACs.

## Supplementary information

**Additional file 1.** The supplementary information includes a complete description of the used noise model. **Table S1.** Optimal sampling schedules for dosimetry based on planar images. **Figure S1.** Variation of the last two time points for *f*_syst_ = 25%. **Figure S2.** Variation of the last two time points for *f*_syst_ = 75%.

## Data Availability

The data underlying the analyses in this manuscript are available on demand from the author.

## Abbreviations

CT: X-ray computed tomography
mCRPC: Metastasized castration-resistant prostate cancer
n/a: Not available
NET: Neuroendocrine tumour
OSS: Optimal sampling schedule
p.i.: Post injection
PBPK: Physiologically based pharmacokinetic
RLT: Radioligand therapy
RMSE: Root-mean-squared error
ROI: Region of interest
SPECT: Single-photon emission computed tomography
TAC: Time-activity curve
TIAC: Time-integrated activity coefficient
TPs: Time points
