# Supplementary material for: Influence of sampling schedules on [177Lu]Lu-PSMA dosimetry
Source: EJNMMI Phys. 2020 Jun 17;7:41. doi: 10.1186/s40658-020-00311-0 (PMC7300169; doi:10.1186/s40658-020-00311-0)
Supplement: Supplementary file 1 — Additional file 1. The supplementary information includes a complete description of the used noise model. Table S1. Optimal sampling schedules for dosimetry based on planar images. Figure S1. Variation of the last two time points for fsyst = 25%. Figure S2. Variation of the last two time points for fsyst = 75%. [file 40658_2020_311_MOESM1_ESM.pdf]

Additional files for:

Influence of sampling schedules on [^{177}Lu]Lu-PSMA dosimetry

Andreas Rinscheid[1,2*], Peter Kletting[1,2], Matthias Eiber[3], Ambros J. Beer[2], Gerhard Glatting[1,2]

[1] Medical Radiation Physics, Department of Nuclear Medicine, Ulm University, 89081 Ulm, Germany
[2] Department of Nuclear Medicine, Ulm University, 89081 Ulm, Germany
[3] Department of Nuclear Medicine, Klinikum Rechts der Isar, Technische Universität München, 81675 München, Germany

*Corresponding author: Andreas Rinscheid, Medical Radiation Physics, Department of Nuclear Medicine, Ulm University, Albert-Einstein-Allee 23, 89081 Ulm, Germany
E-mail: andreas.rinscheid@uni-ulm.de

Table of contents

1 Noise model **2**

2 Optimal sampling schedules for dosimetry based on planar images **3**

3 Variation of the last two time points ($f_{syst} = 25\,\%$) **4**

4 Variation of the last two time points ($f_{syst} = 75\,\%$) **5**

References **6**

1 Noise model

This is a detailed description of the used simulation routine introduced by Rinscheid *et al.* [1]. Activity values $A_{\text{true}}(t)$ taken from the virtual patients were considered as ground truths. Noise following a log-normal distribution was considered for activity values referred to planar images. A log-normal distribution has the probability density function according to [2]

$$f_{\mathcal{LN}}(x,\mu,\sigma) = \frac{1}{x \cdot \sigma\sqrt{2\pi}} \cdot \exp\left(-\frac{1}{2}\left(\frac{\ln(x)-\mu}{\sigma}\right)^2\right), \tag{S1}$$

where μ and σ are the mean and standard deviation of the natural logarithm of $f_{\mathcal{LN}}(x,\mu,\sigma)$. The mean m and standard deviation v of the log-normal distribution itself can be calculated to be

$$m = \mathrm{e}^{\mu+\frac{1}{2}\sigma^2} \tag{S2}$$

$$v = \mathrm{e}^{2\mu+\sigma^2}\left(\mathrm{e}^{\sigma^2}-1\right). \tag{S3}$$

Thus, the parameter μ and σ can be determined from m and v according to

$$\mu = \ln\left(\frac{m^2}{\sqrt{v+m^2}}\right) \tag{S4}$$

$$\sigma = \sqrt{\ln\left(\frac{v}{m^2}+1\right)}. \tag{S5}$$

Drawing a random number x from a log-normal distribution with mean m and variance v is indicated as

$$x \sim \mathcal{LN}(m,v). \tag{S6}$$

The simulated noise for the planar images comprised a systematic (FSD_{syst}) and a stochastic (FSD_{stoch}) part [3], which were successively modelled. First, the systematic noise was randomly selected using the true activity value of the first time point t_1 of the investigated sampling schedule with

$$A_{\text{planar,syst}}(t_1) \sim \mathcal{LN}\left(A_{\text{true}}(t_1), (FSD_{\text{syst}} \cdot A_{\text{true}}(t_1))^2\right), \tag{S7}$$

where $A_{\text{planar,syst}}(t_1)$ is the simulated activity value at time point t_1 considering systematic noise. The relative magnitude of systematic error used for time point t_1 was transferred to the other time points t_i according to

$$A_{\text{planar,syst}}(t_i) = \frac{A_{\text{planar,syst}}(t_1)}{A_{\text{true}}(t_1)} \cdot A_{\text{true}}(t_i). \tag{S8}$$

The stochastic error was individually considered for each time point, i.e. random activity values were drawn from the distribution

$$A_{\text{planar}}(t_i) \sim \mathcal{LN}\left(A_{\text{planar,syst}}(t_i), (FSD_{\text{stoch}} \cdot A_{\text{planar,syst}}(t_i))^2\right), \tag{S9}$$

where $A_{\text{planar}}(t_i)$ are the simulated activity values for planar images considering both systematic and stochastic noise.

The simulated activity value A_{SPECT} attributed to the SPECT/CT measurement needed for applying the hybrid planar/SPECT method was also randomly chosen from a log-normally distributed probability function according to

$$A_{\text{SPECT}}(t_{\text{SPECT}}) \sim \mathcal{LN}\left(A_{\text{true}}(t_{\text{SPECT}}), (FSD_{\text{SPECT}} \cdot A_{\text{true}}(t_{\text{SPECT}}))^2\right), \tag{S10}$$

where t_{SPECT} is the time of the quantitative SPECT/CT measurement, i.e. 0.5 h after a planar image at t_i, and FSD_{SPECT} is the fractional standard deviation attributed to the noise of the SPECT/CT measurement.

The total noise levels for activity values attributed to the SPECT/CT measurement FSD_{SPECT} and to the planar images FSD_{planar} were fixed to 5 % and 20 %, respectively. The noise of the planar images was subdivided in a systematic and a stochastic part according to

$$FSD_{\text{planar}} \approx \sqrt{FSD_{\text{syst}}^2 + FSD_{\text{stoch}}^2}. \tag{S11}$$

Three different fractions of systematic error to the total error of the planar images ($f_{\text{syst}} =$ 25 %, 50 %, 75 %) were investigated.

2 Optimal sampling schedules for dosimetry based on planar images

Table S1: Determined OSS for renal and tumour dosimetry based on 2–4 planar images.

f_{syst} (%)	N_{TP}	OSS (h)	$RMSE_{\text{K}}$ (%)	$\mu_{\text{K}} \pm \sigma_{\text{K}}$ (%)	$RMSE_{\text{T}}$ (%)	$\mu_{\text{T}} \pm \sigma_{\text{T}}$ (%)
25	2	20, 124	17	−0.3 ± 17	18	−2.2 ± 18
50	2	20, 120	18	−0.9 ± 18	19	−2.9 ± 18
75	2	22, 116	19	−1.7 ± 19	20	−4.6 ± 19
25	3	4, 22, 116	15	0.3 ± 15	16	−0.3 ± 16
50	3	20, 28, 192	17	−3.0 ± 17	18	−4.9 ± 18
75	3	20, 76, 92	18	−2.4 ± 18	19	−5.2 ± 19
25	4	4, 20, 44, 192	14	−2.0 ± 14	15	−2.4 ± 15
50	4	4, 22, 44, 192	16	−1.3 ± 16	17	−2.3 ± 17
75	4	4, 28, 68, 144	18	−0.7 ± 18	19	−3.4 ± 18

[a] fraction of systematic error to the total error
[b] number of time points
[c] optimal sampling schedules
[d] root mean squared error of kidneys (subscript "K") and tumours subscript ("T")
[e] mean and standard deviation of the relative deviations of the simulated time-integrated activity coefficients from the ground truth

3 Variation of the last two time points ($f_{\text{syst}} = 25\,\%$)

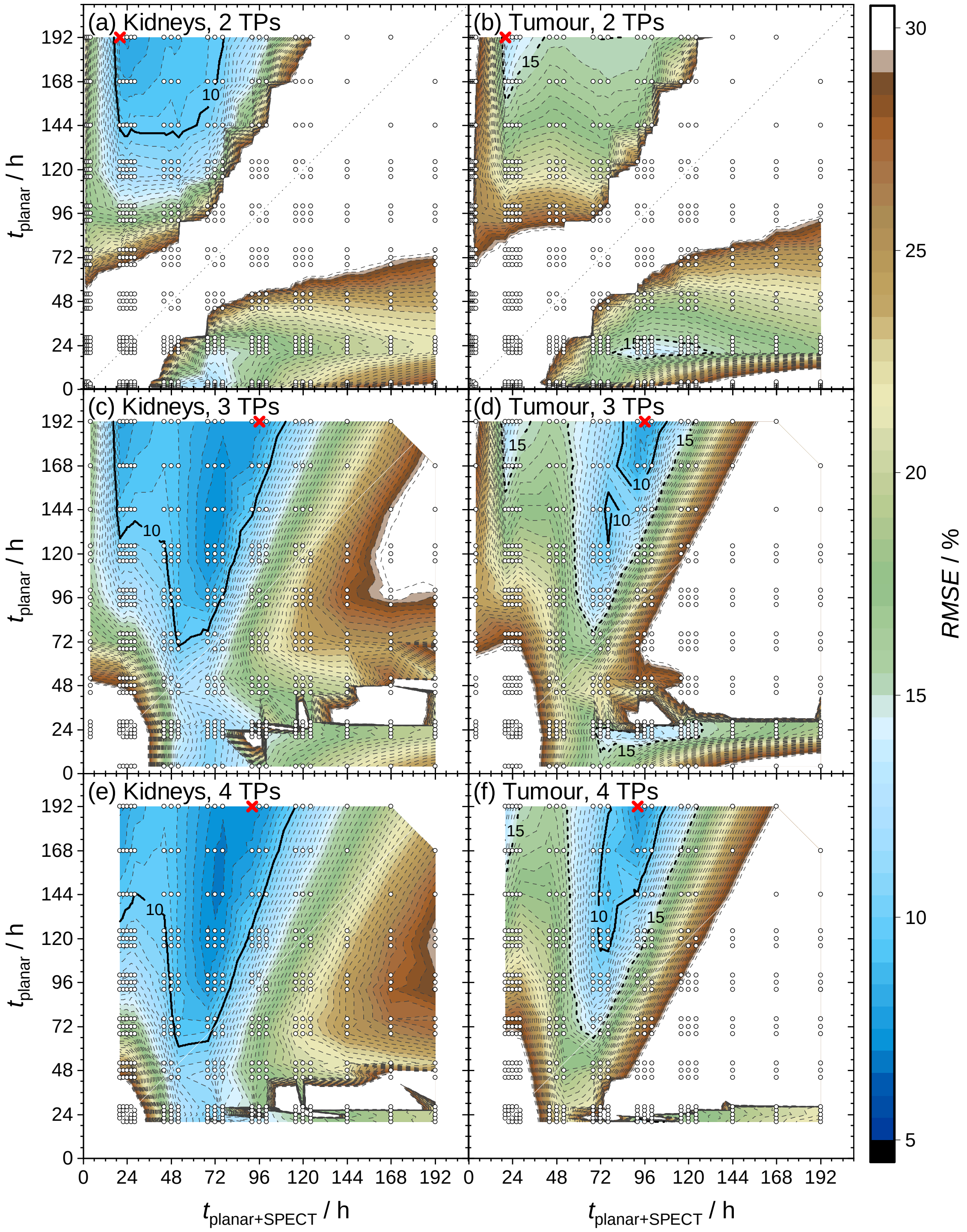

Figure S1: Effect of varying the last two time points (including the time point of the SPECT/CT $t_{\text{planar+SPECT}}$) from the optimal schedules for the hybrid planar/SPECT method (Table 1 in the manuscript) on the root-mean-square error values ($RMSE$) of the kidneys and the tumours. The optimal schedules and the investigated alternatives of the last two time points are marked with red crosses and white circles, respectively. Contour lines with $RMSE = 10\,\%$ for the kidneys (black line) and with $RMSE = 10\,\%, 15\,\%$ (black dashed line) for the tumours are highlighted. The results base on the simulations using a fraction of systematic error to the total error of $f_{\text{syst}} = 25\,\%$.

4 Variation of the last two time points ($f_{\text{syst}} = 75\,\%$)

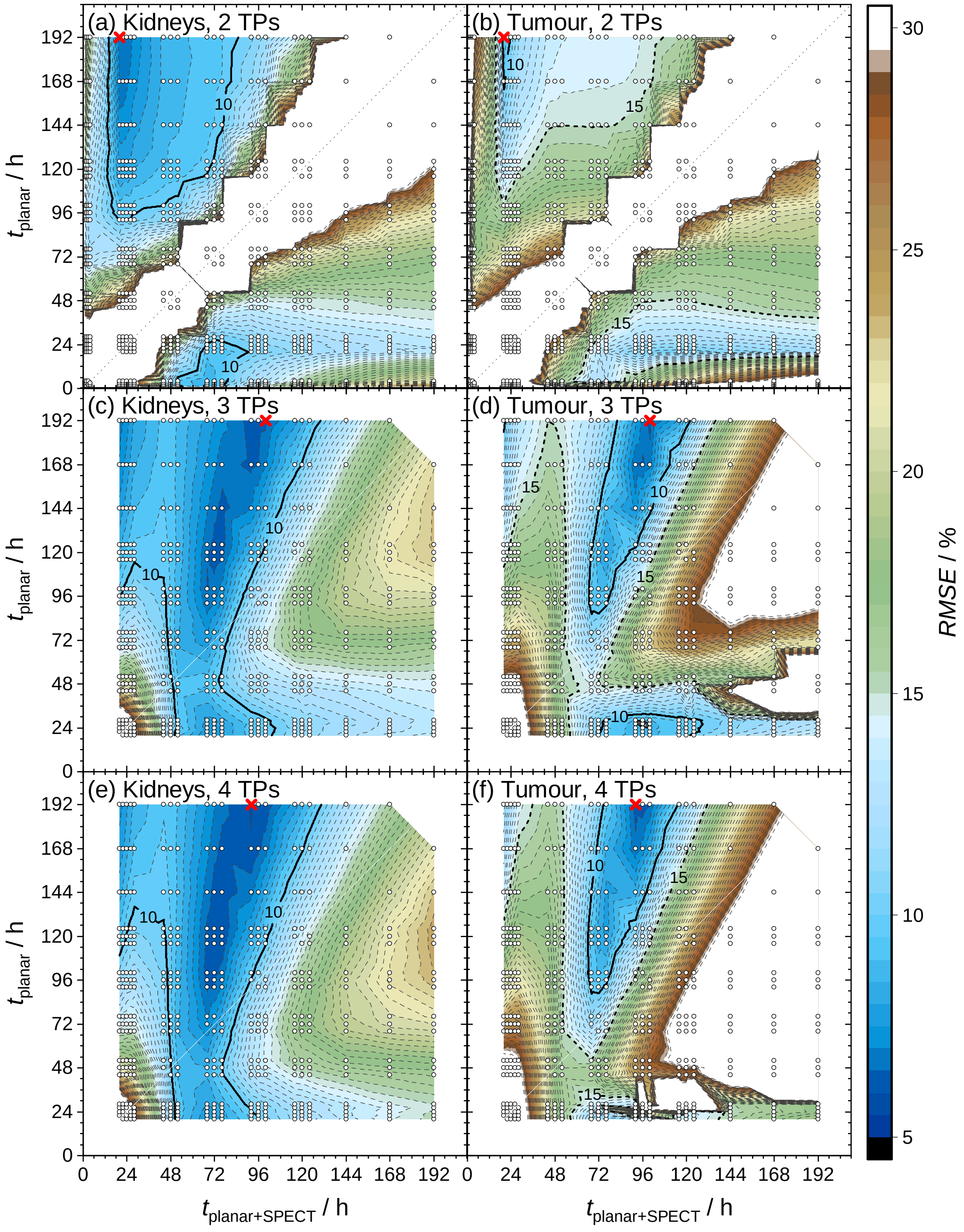

Figure S2: Effect of varying the last two time points (including the time point of the SPECT/CT $t_{\text{planar+SPECT}}$) from the optimal schedules for the hybrid planar/SPECT method (Table 1 in the manuscript) on the root-mean-square error values ($RMSE$) of the kidneys and the tumours. The optimal schedules and the investigated alternatives of the last two time points are marked with red crosses and white circles, respectively. Contour lines with $RMSE = 10\,\%$ for the kidneys (black line) and with $RMSE = 10\,\%, 15\,\%$ (black dashed line) for the tumours are highlighted. The results base on the simulations using a fraction of systematic error to the total error of $f_{\text{syst}} = 75\,\%$.

References

1. Rinscheid A, Kletting P, Eiber M, Beer AJ, and Glatting G. Technical Note: Optimal sampling schedules for kidney dosimetry based on the hybrid planar/SPECT method in ^{177}Lu-PSMA therapy. Med Phys 2019;46:5861–5866.
2. Limpert E, Stahel WA, and Abbt M. Log-normal Distributions across the Sciences: Keys and Clues. BioScience 2001;51:341–352.
3. Gear JI, Cox MG, Gustafsson J, Sjögreen-Gleisner K, Murray I, Glatting G, Konijnenberg M, and Flux GD. EANM practical guidance on uncertainty analysis for molecular radiotherapy absorbed dose calculations. Eur J Nucl Med Mol Imaging 2018;45:2456–2474.
